# *Salvia officinalis* L. Essential Oil: Characterization, Antioxidant Properties, and the Effects of Aromatherapy in Adult Patients

**DOI:** 10.3390/antiox11050808

**Published:** 2022-04-21

**Authors:** Maria-Daniela Mot, Simona Gavrilaș, Andreea I. Lupitu, Cristian Moisa, Dorina Chambre, Delia Mirela Tit, Mihaela Alexandra Bogdan, Adina-Maria Bodescu, Lucian Copolovici, Dana Maria Copolovici, Simona Gabriela Bungau

**Affiliations:** 1 Doctoral School of Biomedical Sciences, University of Oradea, 410087 Oradea, Romania; mariadanielamot@gmail.com (M.-D.M.); dtit@uoradea.ro (D.M.T.); mihaela.alexandra.bogdan@gmail.com (M.A.B.); sbungau@uoradea.ro (S.G.B.); 2 Faculty of Food Engineering, Tourism and Environmental Protection, Institute for Research, Development and Innovation in Technical and Natural Sciences, “Aurel Vlaicu” University of Arad, 310330 Arad, Romania; simona.gavrilas@uav.ro (S.G.); pag.andreea@yahoo.com (A.I.L.); moisa.cristian@yahoo.com (C.M.); dorinachambree@yahoo.com (D.C.); lucian.copolovici@uav.ro (L.C.); 3 Department of Pharmacy, Faculty of Medicine and Pharmacy, University of Oradea, 410028 Oradea, Romania; 4 “Adam Müller Guttenbrunn” High School, 310245 Arad, Romania; adina.bodescu@ltamg.ro

**Keywords:** *Salvia officinalis*, essential oil, chemical analysis, antioxidant activity, aromatherapy, inhalation

## Abstract

The purpose of this study is to reveal the chemical and biochemical characteristics and the potential aromatherapy applications of the essential oil (EO) of *Salvia officinalis* (common sage) within a hospital environment. The chemical composition was determined by gas chromatography with mass spectrometry and ATR-FTIR spectroscopy. Three types of sage EOs were included in this study: two commercial oils and one oil obtained by in-house hydrodistillation. Based on the findings, these EOs were included in different chemotypes. The first two samples were similar to the most common chemotype (α-thujone > camphor > 1,8-cineole > β-thujone), while the in-house sage EO revealed a high content of 1,8-cineole, borneol, α-thujone, similar to the Dalmatian type. The latter sample was selected to be evaluated for its antioxidant and medical effects, as borneol, a bicyclic monoterpene, is known as a substance with anesthetic and analgesic effects in traditional Asian medicine. The study suggests that the antioxidant capacity of the sage EO is modest (33.61% and 84.50% inhibition was determined by DPPH and ABTS assays, respectively), but also that the inhalation of sage EO with high borneol content by hospitalized patients could improve these patients’ satisfaction.

## 1. Introduction

Essential oils (EOs) have proven their specific applications in different areas, including in human health, as a treatment for various disorders (migraine [1], skin disorders [2], fatigue [3,4], stroke [5], sleep disorders [6,7], endocrine disorders [8], depressive disorders [9], etc.). In medicine, EOs are considered good candidates as complementary and alternative treatment components due to their antimicrobial properties [10], possible anesthetic [11], or immunostimulatory effects [12]. An EO can be used alone and/or in synergetic associations with other EOs [13,14].

The qualitative composition of essential oils is determined by the genome of the particular plant and the variation caused by several environmental conditions such as daylight, temperature, and light. The conditions mentioned above select a pattern of seasonal fluctuations in plant metabolites. These conditions vary predictably and significantly throughout the vegetation interval, usually recurrent each year [15].

*Salvia officinalis* L. is one of the most widely used sources for EOs in traditional medicine. Even though the chemical composition of *Salvia officinalis* EOs has already been determined, the composition of EOs is very complex, depending on the plant part, time of harvesting, season, genetic diversity, climate, and meteorological conditions. Different studies have used gas-chromatography techniques [16,17,18,19] or FTIR [20] to determine *Salvia officinalis* L. EOs’ chemical composition. It is claimed that the antimicrobial properties of EOs are related to an extended alcoholic profile. Also, it was assessed that skin injury restoration is enhanced in the case of using plant extracts containing increased amounts of ketones [2,21]. EOs are rapidly absorbed via the skin into the bloodstream and thus travel to the brain and other organs [1,4,5,9,22,23,24,25,26,27,28]. A reduction of migraine discomfort is the result of the combination of the following factors: neurogenic inflammation, as well as a decrease in distress perception, along with blood vessel dilation [1]. The same processes may be responsible for the positive response to aromatherapy and synergic musical therapy obtained for anxiousness and/or suffering states recorded in different post-procedural phases of adult patients [29,30]. For insomnia improvement, the olfactory route is considered based on the comparative results regarding inhalation versus the transdermal pathway [31]. The inhalation of EOs may stimulate the immune and limbic systems responsible for the body’s state of well-being and emotional integration. The quality of life in burnout cases, determined by different diseases, such as chronic hemodialysis [32] or long periods of hard work, could be improved through aromatherapy [33,34]. Several reports are focused on evaluating essential oils’ toxicity profile [35,36,37]. For example, sage essential oil toxicity has been assessed on rat hepatocytes [36], and it is not toxic at concentrations below 200 nL mL^−1^. A report presents an accidental exposure to a newborn and a toddler to sage EO that lead to generalized tonic-clonic seizures [38]. Not all medical aspects are fully elucidated so far, as relevant elements such as the toxicological profile and different interactions still need to be clarified and studied.

It is used to treat colds, tuberculosis, bronchitis, gastrointestinal diseases, inflammation, and presents antibacterial, antifungal [39], antitumor [37,40,41,42], and antioxidant properties [43]. With respect to *Salvia officinalis,* different mechanisms of action have been proposed. Most of the research has been focused on the influence of *Salvia officinalis* on mental functions. Memory enhancement is thought to be a consequence of *Salvia officinalis*’ stimulating effect on the nicotinic and muscarinic receptors. Studies have also been conducted on the inhibitory effect on acetylcholinesterase, a compound considered to have a major role in Alzheimer-specific symptom progress [44]. At 100 mg/mL concentration, *Salvia officinalis* extract revealed high activity towards *Escherichia coli*, which is ordinary with respect to *Staphylococcus aureus* and *Pseudomonas aeruginosa*, and low in the case of *Bacillus subtilis* [45]. The use of the extracts in the presence of Hp-2-Minh ligand improved their biocidal characteristic even at lower concentrations. Research has proven the antimicrobial potential of EOs. This finding has medical significance, as it justifies reducing the use of synthetic medicinal products and utilizing EOs as a viable alternative in cases of drug-resistant pathogens.

Aromatherapy refers to the use of volatile compounds or fragrances from EOs obtained from plants, typically by inhalation, to prevent or cure diseases, infections, and indispositions. For alleviating pain, nausea, and anxiety, an ancient practice—clinical aromatherapy—is gaining attention in contemporary health services, where the intention of both the consumers and clinicians is to minimize the usage of medications. Thus, using integrative and complementary therapies is increasing, and scientific proof for the integrative therapies continues to flourish [46].

Different EOs were included in complementary and alternative treatment (such as aromatherapy) and were evaluated in a hospital setting in the following instances: peppermint EO for nausea and vomiting in women [25], lavender EO [47], *Salvia officinalis* EO for reducing nausea and vomiting in patients with cancer undergoing chemotherapy [48], *Rosa damascene* EO to decrease anxiety and increase sleep quality in cardiac patients [49], and *Citrus aurantium* to reduce anxiety in patients with acute coronary syndrome [50]. In the case of hospitalized patients, one of the main issues that arise is the decrease of peace of mind and the increase in anxiety during the hospitalization period [51].

Therefore, the present investigation aimed to assess the chemical composition and antioxidant activity of the essential oil of *S. officinalis* obtained from aerial parts of the plant and evaluate aromatherapy’s influence on patients in a hospital setup. The results of this study can be a starting point for the development of Aromatherapy Programs in Patient Care Settings in Romania.

## 2. Materials and Methods

All the reagents and solvents used in the experimental part were of adequate analytical or chromatographical grade (Sigma Aldrich, Fluka, Switzerland, and Merck, Darmstadt, Germany).

### 2.1. Sample Collection and Preparation

The EO named L-SEO was obtained by hydro-distillation from the dried aerial parts of *Salvia officinalis* plants grown in Arad County, Romania (coordinates 46°10′30″ N 21°18′45″ E, sample harvested in June 2019). The EO was stored in glass vials at +4 °C until further analysis. The samples B-SEO and EG-SEO are sage EOs commercially available on the Romanian market.

### 2.2. GC-MS Determination of the Chemical Composition of the Essential Oil from Salvia officinalis

The constituents of the EOs were determined by the gas chromatography method, using a gas chromatograph (GC) (Shimadzu2010, Kyoto, Japan) coupled with a triple quadruple mass spectrometer (MS) (TQ 8040, Shimadzu, Kyoto, Japan). The column used was an Optima 1 MS (30 m × 0.25 mm i.d., with a film thickness of 0.25 mm). At a flow rate of 1 mL/min, Helium was used as a carrier gas. The procedure for separating and quantifying EO components is described above [52]. The compounds from the analyzed samples were identified based on their mass spectra using the NIST 14 and Wiley 09 mass spectrum libraries (Scientific Instrument Services, Palmer, MA, USA). The Kovats retention indices for the identified compounds were calculated using the C8-C40 alkane standard. All analyses were performed in triplicate.

### 2.3. ATR-FTIR Spectroscopy

The ATR-FTIR spectra of *Salvia officinalis* L. EOs (L-SEO, B-SEO, and EG-SEO) were obtained on the wavelength range between 600 and 4000 cm^−1^ with a Bruker Vertex 70 (Bremen, Germany) spectrophotometer equipped with a Pike Miracle ATR cell. A sample volume of ~10 µL from each EO was placed directly on the surface of the ZnSe ATR crystal in the Teflon depression and covered with a metal cover. To avoid evaporation of the sample, a black outer ring was screwed on to press the Teflon depression tightly on the crystal. At the same time, the upper handle of the ATR cell was rotated so that its slightly concave stainless steel tip applied pressure against the metal cover.

The experimental spectra of the samples were recorded with a resolution of 4 cm^−1^, and 32 scans were accumulated per spectrum. The spectra were obtained in duplicate for each sample, and the average spectrum of the two measurements was calculated. Before each ATR measurement, the ZnSe crystal was carefully cleaned with isopropyl alcohol, and an air background spectrum was performed.

The OPUS software, version 6.5 from Bruker (Germany), was employed for spectra acquisition, minimum–maximum normalization, baseline correction, and identifying the wavelength value corresponding to the maximum absorbance of the recorded FT-IR bands.

### 2.4. Antioxidant Activity (DPPH and ABTS Assays)

The L-SEO sample’s antioxidant capacity was evaluated using two spectrophotometric assays (DPPH assay and ABTS assay), as reported earlier [22]. A UV-VIS spectrophotometer Specord 200 (Analytik Jena, Jena, Germany) and a 10 mm quartz cuvette were used in the procedure described above. Briefly, for the DPPH assay, we mixed a 0.1 mL control sample with 3 mL of 0.2 mM ethanolic DPPH• solution. The absorbance was recorded after 60 min of reaction in the dark at λ = 517 nm. Positive controls containing 0.02–4 mM Trolox solutions were prepared. The data are expressed in mg Trolox/L and inhibition (%).

For the ABTS assay, we mixed 0.5 mL sample or control with 1 mL ABTS* solution, prepared 16 h before from ABTS reagent and 2.45 mM aqueous solution of sodium persulfate. The absorbance was recorded at λ = 734 nm, after 10 min of reaction time in the dark. Positive controls containing 0.02–1.0 mM Trolox solutions were prepared. The data are expressed in mmol TEAC/L (TEAC: Trolox Equivalent Antioxidant Capacities) and inhibition (%). All the experiments were performed in triplicate.

### 2.5. Aromatherapy Effects: Clinical Application

This study was performed between August 2019 and August 2020, as a randomized, single-blind study involving adult patients at the Lipova City Hospital, Arad County, Romania.

In total, 174 hospitalized patients aged 23–85 years were enrolled, meeting the following inclusion criteria: adult patients with chronic conditions who have had at least one hospitalization in the same section in the past, an unimpaired sense of smell, no psychiatric pathologies, ability to communicate, and minimum 4 days and maximum 5 days hospital stay. Exclusion criteria included surgery intervention and unwillingness to participate.

Patients were randomized into two groups: 50 in the control group and 124 in the aromatherapy group; no patients were excluded, and no patients quit the study. Random allocation of the patients was made by hospital personnel who were not involved in the data collection or data analysis, depending on the number of patients hospitalized in each room. During the hospitalization period, patients admitted to the same ward were included in either the aromatherapy group or the control group (there were no patients from both groups at the same time in a ward). For the patients enrolled in the aromatherapy group, on a daily basis, a hospital staff member prepared the cotton disc with two drops of sage EO (L-SEO sample) that was kept on the patient’s pillow for a minimum of 30 min as the patient inhaled the volatile compounds. The patients from the control group received only routine care.

Only one EO (L-SEO) was used in aromatherapy, which was selected after determining the chemical composition.

Descriptive information form: A questionnaire was prepared by the authors of this study, including the patients’ related characteristics such as gender, age, weight, educational level, habits, personal evaluation, and health status. The questionnaire was completed by respondents on the last day of the hospitalization and was used to determine the following: (a) the demographic profile of the participants in terms of age, gender, weight, educational level, social status, health status in the last year, marital status, the use of aroma indoor and of perfumes, the use of sedatives/anxiolytics and the presence of some allergies; (b) the evaluation on the quality of in-hospital services in the present hospitalization stay, with seven possible answers: very weak, weak, acceptable, improved, good, very good, and excellent.

The study was approved by the Ethics Committee of Lipova City Hospital (no. 62/1 July 2019) and was conducted following the ethical principles of the Declaration of Helsinki [53]. The patients provided their written informed consent after a verbal and written explanation of the study protocol.

### 2.6. Statistical Analysis

Data were processed via GraphPad Prism (version 5.0 for Windows, GraphPad Software, San Diego, CA, USA), and F values (at *p* < 0.05) were considered statistically significant. The *t*-test, chi-squared test, Kruskal-Wallis test, Fisher’s exact test, Friedman test, and the Mann-Whitney test were used to compare qualitative variables in two groups.

## 3. Results

### 3.1. Chemical Analyses

#### 3.1.1. GC-MS Analyses

**Figure 1:**
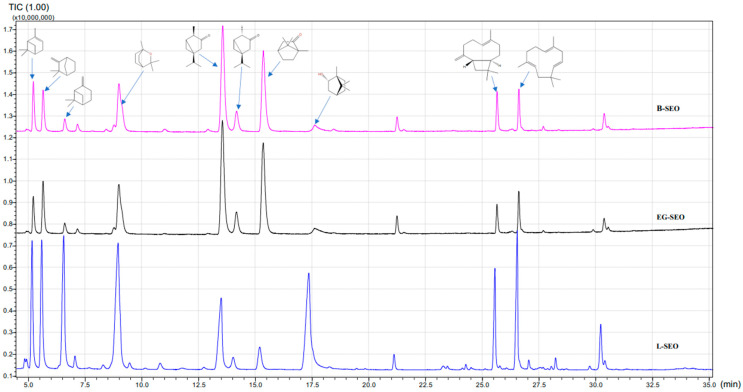
GC-MS chromatograms obtained for the investigated *Salvia officinalis* essential oil samples: B-SEO, pink; EG-SEO, black; L-SEO, blue.

Figure 1 depicts the chromatograms obtained for the three *Salvia officinalis* essential oils samples investigated in this study, and Table 1 shows their composition as determined by GC-MS method. The EOs’ constitutive elements are listed according to the elution time. In the liquid phase, 47 compounds were separated.

The results obtained follow the data presented in the literature [43]. The monoterpenes are the most represented, whilst the differences between the hydrocarbons (1,8-cineole, 17.98%; β-pinene, 10.52%; camphene, 8.73%) and the oxygenated (borneol, 15.86%; β-thujone, 1.34%) ones are modest. The sesquiterpenes have a prevalence lower than 9% (α-humulene, 8.64%; β-caryophyllene, 5.66%; caryophyllene oxide, 0.22%). The analyzed samples have a different composition in comparison to major compounds. B-SEO and EG-SEO have two major compounds (namely α-thujone and camphor), whilst L-SEO has four major compounds (namely 1,8-cineol, borneol, β-pinene, and α-thujone). L-SEO has a closer composition to the marocain one, possibly due to the plant growing conditions. B-SEO and EG-SEO have a composition similar to the EO obtained in Turkey, as shown in Table 1.

The differences in the chemical composition of the analyzed EOs could be attributed to several factors such as environmental factors, growth conditions, and time of harvesting [19,43,54]. The results obtained in this present study agree with previous research that concluded that 1,8-cineole, camphor, and α- and β-thujone are the main components of *Salvia officinalis* EOs [17,18,19,37]. As shown earlier, (+)-borneol is the only enantiomer found in the EO of Salvia species [55]; this compound is associated with analgesic and sedative effects [56].

#### 3.1.2. ATR-FTIR Spectroscopy

**Figure 2:**
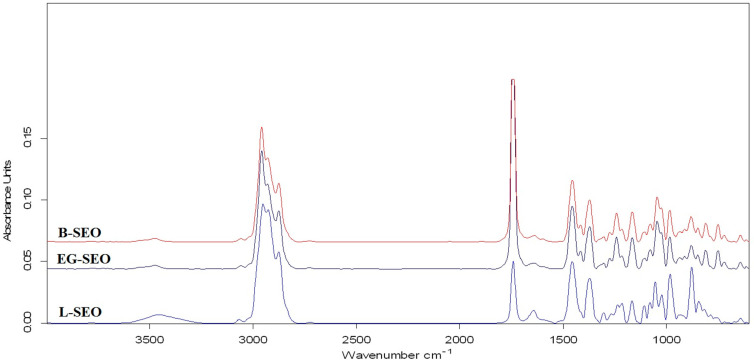
The ATR-FTIR spectra of the *Salvia officinalis* L. essential oil samples.

The obtained Attenuated Total Reflectance-Fourier Transform Infrared (ATR-FTIR) spectra for the investigated *Salvia officinalis* L. EOs are depicted in Figure 2, and the wavelengths values (in the 600–4000 cm^−1^ range) for all recorded peaks are presented in Table 2. The different chemical compositions of the investigated EOs samples led to obtaining major differences in the intensity of the peaks located at the following wavelengths (L-SEO/B-SEO/ED-SEO): 3459/3474/3474 cm^−1^, 1741/1741/1741 cm^−1^, 1642/1637/1638 cm^−1^, 1054/-/- cm^−1^, -/1045/1045 cm^−1^, 982/983/983 cm^−1^, and 877/880/879 cm^−1^.

#### 3.1.3. Antioxidant Capacity

The antioxidant capacity of the sage EO (sample L-SEO) was evaluated by using two methods: DPPH assay (inhibition 33.61 ± 2.12%, antioxidant activity 0.81 ± 0.11 mg Trolox/L) and ABTS assay (inhibition 84.50 ± 2.23%, antioxidant activity 0.81 ± 0.03 mmol TEAC/L). All analyses were performed in triplicate. The data are expressed as mean ± STDEV.

### 3.2. Patients’ Characteristics

The mean age of the patients from the control group was 58.74 years whilst that of the salvia EO group was 52.34 years, with a *p*-value of 0.3530 (*t*-test). There are no significant differences between the two groups with respect to the following characteristics: gender, weight, educational level, social status, health status in the last year, marital status, the use of aroma indoor and perfumes, the use of sedatives/anxiolytics, and the presence of some allergies (Table 3). Between the groups, there is a significant difference in the variables of the consumption of alcohol, smoking, and the presence of chronic disease.

**Figure 3:**
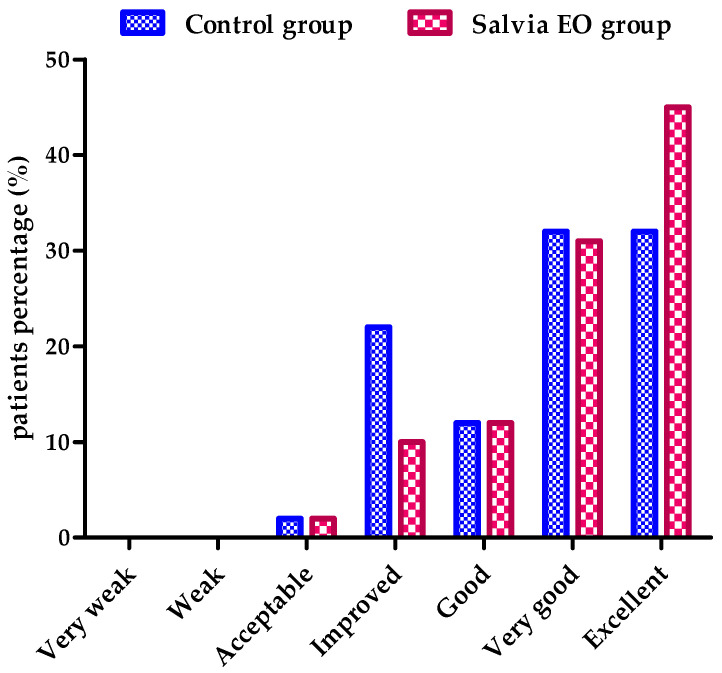
Patients’ satisfaction with the treatment they received during their current hospitalization (Mann-Whitney U test).

The patients’ perception of the quality of in-hospital services and, thus, their well-being after receiving treatment while hospitalized were assessed by answering questions designed with seven possible answers: very weak, weak, acceptable, improved, good, very good, and excellent. Group comparison was made using the Mann-Whitney U test. No significant differences were observed in the between-group comparison (*p* = 0.8969, U = 23). Most patients in both groups rated in-hospital services as excellent and very good (64% in the control group and 76% in the aromatherapy group) (Figure 3). No adverse effects were reported by patients or observed by medical staff in this study.

## 4. Discussion

Much research has focused on investigating sage EO extracted from sage leaves harvested from diverse parts of the world. The percentage of the constituents varies widely due to geographical region, season, environmental conditions, genetic differences, phenological stages, sampling, and extraction methods. The number of detected constituents varies, being around 14–67 (14 with concentrations > 1% [41], 15 [62], 22 [17], 23 [43], 42 [19], 44 [63], 67 [64]). Also, sage EO’s composition is dependent on the growing ecosystem conditions, as determined in an exhaustive study reported by Russo et al., where 18 samples were collected in 2008–2009 in the south-central part of Italy and investigated [41]. The EOs obtained from sage leaves contained α-thujone (7.8–20.1%), camphor (8.4–20.8%), borneol (2.5–16.9%), γ-murolene (2.9–13.8%), and sclareol (5.9–23.1%) as the major compounds. Recently, another study identified oxygenated monoterpenes (67.7%) and monoterpenes hydrocarbons (19.1%) in the EO from sage leaves harvested in June 2020 in Tuscany, Italy. The main components identified in this EO batch were 1,8-cineole (30.3%), camphor (17.1%), α-thujone (9.7%), camphene (7.9%), and chrysanthenone (6.8%) [43].

Due to their chemical composition (shown in Table 1), two of the investigated samples (B-SEO and EG-SEO) were included in the more common C1c chemotype, as classified by Craft et al. [65]: α-thujone > camphor > 1,8-cineole > β-thujone, which was reported in samples from Turkey [17], Brazil [16], Mexico [65], and Croatia [66]. Meanwhile, the L-SEO sample is a chemotype found in the sage EO from the Dalmatian region, where 1,8-cineole is the main component and borneol is in high quantity [67].

The reported FTIR results of the EOs show that the components found in higher concentrations dominate the resulting vibrational spectra, while the components found in low concentrations do not have significant influence [54,68,69]. Ciko et al. [20] showed that FTIR analysis of the EO obtained from *Salvia officinalis* L. leaves indicated the presence of monoterpenes such as thujones, camphor, 1,8-cineole, and pinene.

The GC-MS results (Table 1) obtained for L-SEO sample indicate 1,8-cineol (17.98%) and borneol (15.86%) as their main components, which can influence the features of the recorded ATR-FTIR spectrum and the intensity of the obtained bands (Figure 2). The 1,8-cineole (eucalyptol) can be distinguished by the following key bands: 1371 cm^−1^ (δ_sym_(CH_3_-CO) symmetric in plan bending/scissoring), 1266 cm^−1^ (ν(C-O) stretching from alkyl ether, 1214 cm^−1^ (ν_asym_(C-O-C) asymmetric stretching), 1080 cm^−1^ (ν_sym_(C-O-C) symmetric stretching), and 982 cm^−1^ (ω_asym_(CH_2_) asymmetric out-of-plane bending/wagging vibration). Due to the higher content of 1,8-cineole in L-SEO, the 982 cm^−1^ band is more intense than in B-SEO and EG-SEO, with 844 cm^−1^ corresponding to ω_asym_(C-H) asymmetric out-of-plane bending/wagging vibration [20,70,71].

For borneol, the characteristics bands are 3459 cm^−1^ (ν_sym_(O-H) stretching from H-bonded alcohols), 1303 cm^−1^ (δ_sym_(O-H) scissoring vibration), 1166 cm^−1^ (δ_sym_(C-OH) in-plane bending of alcohol groups), 1054 cm^−1^ (ν_asym_(C-O) asymmetric stretching from primary alcohols), 1106 cm^−1^ (ν_sym_(C-O) symmetric stretching deformation), and 642 cm^−1^ (ω(C-O) out-of-plane bending) [20,30].

Other classes of compounds that occur in high proportions in the composition of L-SEO are bicyclic monoterpenes with =CH_2_ alkyl groups and sesquiterpenes with C=C bonds (sabinene + camphene, 8.87%; β-caryophyllene + α-humulene, 14.3%), with the key bands located at 3066 cm^−1^ (ν_sym_(=C-H), Csp^2^ from the terminal vinyl group) and 1642 cm^−1^ (ν(C=CH_2_) stretching vibration). Schultz et. al. reported a band due to the stretching vibration of (C=C) located at 1653 cm^−1^ for sabinene and 1635 cm^−1^ for β-caryophyllene [70], while Agatonovik-Kustin et al. mentioned a vinyl group vibration at 1635–1650 cm^−1^, although the intensity of this peak was very low [70].

Compared to B-SEO and EG-SEO samples, in the case of L-SEO, the 1642 cm^−1^ band is more evident due to the higher content of compounds with =CH_2_ and C=C groups, with 1456 cm^−1^ corresponding to δ_sym_(C-H) and δ_asym_(C-H) in-plane bending of CH_3_ and -CH_2_ groups. The C=CH_2_ in-plane deformation vibration was not recorded as a separate band near ~1410–1420 cm^−1^ [20], probably being hidden under the -CH_3_ and -CH_2_ absorption bands, with 877 cm^−1^ corresponding to ν(=CH_2_) methylene out-of-plane deformation due to the strained ring structure with an exocyclic =CH_2_ group [70]. The higher intensity of the 877 cm^−1^ band, for the L-SEO compared with B-SEO and EG-ESO, is due to the higher content of compounds with the exocyclic =CH_2_ group.

In addition, it is mentioned that in the 2850–3000 cm^−1^ wavelength range, a group of characteristic bands with strong intensity were recorded (2951 cm^−1^, 2926 cm^−1^, 2875 cm^−1^) due to the symmetric and asymmetric stretching vibration of (C-H) bond from CH_3_, CH_2_, and CH. The intensities of these bands are higher for L-SEO than for B-SEO and EG-SEO.

For the B-SEO and EG-SEO samples, the obtained GC-MS results (Table 2) reveal that thujone and (+) camphor are the major components (50.77%) that influence the absorption band intensity. These compounds are monoterpenes with a carbonyl group (>C=O), belonging to the ketones class. In the obtained B-SEO and EG-SEO ATR-FTIR spectra (Figure 3), the band positioned at 1741 cm^−1^ is attributed to thujones and camphor being specific for (ν(C=O)) stretching vibration of 5-membered cyclic ketones [18,23]. Compared with the L-SEO sample, in the case of B-SEO and EG-SEO, the intensity of the 1741 cm^−1^ band is higher due to the different chemical compositions of the EOs.

Other absorption bands that showed changes in intensity compared to L-SEO sample, due to the difference in the chemical composition of B-SEO and EG-SEO, were observed at 3474 cm^−1^ due to (ν(O-H)) stretching vibration, weak band; 1415 cm^−1^ due to (ν(C=CH_2_) in-plane deformation vibration; 1241 cm^−1^ due to (ν(C-O)) stretching vibration; and 1045 cm^−1^ due to (ν(C-O)) stretching vibration from primary alcohols [69].

The main efficient constituents of *Salvia essential* oils are terpenes and polyphenols, with one major bioactivity being represented by their antioxidant action. The antioxidant capacity of the investigated sage EO (L-SEO) reached 33.61% and 84.50% by DPPH and ABTS analyses, respectively, which are values specific to samples rich in 1,8-cineole (60–70% inhibition in [69,70,71,72]).

The use of plant-derived essential oils for wellness and to sustain human health is called aromatherapy. In British herbal encyclopedias, the species of the Salvia family have long been renowned for being characterized as compounds that improve memory. Salvia essential oils can be helpful for the enhancement of cognition and mood, as demonstrated by various studies [73].

In this randomized, controlled study, the participants were adult men and women undergoing hospitalization who received or did not receive EOs for daily inhalation. The demographic and clinical data (shown in Table 3) revealed no statistical differences between the groups.

Our results revealed that the level of patient perception of the quality of in-hospital services, when sage EO is inhaled daily by patients, during hospitalization, is not statistically significant, as compared to that of the patients from the control group. The aromatherapy confirmed its beneficial action as a complementary treatment to increase the well-being of patients during hospitalization, as it showed a significant effect on hospitalized patients who evaluated their level of satisfaction as excellent (Figure 3).

The main limitations of this study consist of the somewhat limited number of patients in each group and a lack of medium/long-term follow-up. Further studies with a more extended follow-up period and more in-depth research on the use of sage EO in massage are needed to confirm our findings. Additionally, it is worth noting that the study did not include post-surgery patients. Therefore, our future goal is to develop randomized clinical trials to evaluate the large-scale efficacy of aromatherapy as an alternative therapy in patients with diverse pathologies. On the other hand, some strengths of this study must be highlighted, including the use of a chemically characterized EO in aromatherapy and the increase in some of the patients’ satisfaction regarding their hospitalization experience with this approach.

## 5. Conclusions

The GC-MS analysis of three samples of *Salvia officinalis* L. EOs indicated the presence of terpenes such as 1,8-cineole, thujones, borneol, camphor, sabinene, camphene, and caryophyllenes as the main components. The FTIR results agree with the GS-MS data of investigated samples, which leads to the conclusion that two commercial samples are part of the α-thujone > camphor > 1,8-cineole > β-thujone chemotype. In contrast, the other, in-house sample is part of the 1,8-cineole, borneol chemotype (Dalmatian type). The chemical analysis of EOs used in aromatherapy mechanism is very important, as different biological outcomes could arise based on the major compounds identified. The present results did not reveal a statistically significant difference between groups that inhaled sage EO during hospitalization versus the group that did not. However, more research is needed to investigate the mechanisms responsible for specific effects attributed to EOs to justify the therapeutic effect of sage EO and identify the side effects related to this product.

## Figures and Tables

**Table 1:** *Salvia officinalis* L. essential oil chemical composition (% TIC) determined by GC-MS analyses.

No.	KI	Compound/Class	Present Study			Turkey [17]	Poland [18]	Morocco [37]	Sudan [19]	Brazil [16]
			B-SEO	EG-SEO	L-SEO					
1	925	Unidentified	0.21 ± 0.01	0.24 ± 0.01						
2	926	Tricyclene/MH			0.42 ± 0.01					
3	930	α-Thujene/MH	0.17 ± 0.01	0.15 ± 0.01	0.45 ± 0.01			0.31		
4	939	α-Pinene/MH	5.96 ± 0.03	4.23 ± 0.02	7.8 ± 0.05	7.17	0.02	3.18	8.96	3.07
5	954	Camphene/MH	5.59 ± 0.07	6.92 ± 0.10	8.73 ± 0.12	8.40		3.67	5.09	4.40
6	975	Sabinene/MH	1.70 ± 0.01	1.55 ± 0.03	0.14 ± 0.01			0.41	0.15	
7	979	β-Pinene/MH	0.78 ± 0.03	0.55 ± 0.02	10.52 ± 0.15	2.92	0.40	2.57		
8	990	β-Myrcene/MH			0.72 ± 0.06	1.16		1.94	3.65	
9	1012	4-Carene/MH	0.42 ± 0.02	0.07 ± 0.01						
10	1024	p-Cymene/MH	0.85 ± 0.02	1.01 ± 0.03		1.33				
11		α-Terpinene/MH			0.34 ± 0.02	0.18		0.2	0.22	
12	1029	1,8-Cineole/MH	13.39 ± 0.21	14.22 ± 0.19	17.98 ± 0.23	18.54	23.72	17.52		14.8
13	1050	trans-β-Ocimene/MH			0.52 ± 0.06					
14	1051	D-Limonene/MH				2.46		1.7	0.37	
15	1056	Linalool/MH							0.79	
16	1059	γ-Terpinene/MH	0.60 ± 0.05	0.15 ± 0.06	0.62 ± 0.08	0.18		0.42		
17	1088	α-Terpinolene/MH	0.60 ± 0.10	0.20 ± 0.05		0.12				
18	1089	2-Carene/MH			0.19 ± 0.01					
19	1102	α-Thujone/MO	26.03 ± 0.25	26.73 ± 0.26	8.74 ± 0.12	22.30		21.22	0.91	24.8
20	1109	β-Thujone/MO	4.65 ± 0.11	4.19 ± 0.12	1.34 ± 0.11	14.28		13.45		3.97
21	1146	Camphor/MO	20.09 ± 0.24	22.64 ± 0.27	2.56 ± 0.12	14.40	18.22	21.23	11.57	10.9
22	1169	Borneol/MO	3.1 ± 0.20	3.31 ± 0.19	15.86 ± 0.23	0.37	2.42	1.67	0.81	11.1
23	1188	α-Terpineol/MO	0.50 ± 0.07	0.46 ± 0.08						
24	1287	Bornyl acetate/MO	1.93 ± 0.11	2.27 ± 0.13	0.88 ± 0.06	0.32	0.22			
25	1290	Thymol/MO			0.33 ± 0.01					
26	1351	α-Cubebene/SH			0.2 ± 0.06					
27	1380	α-Copaene/SH			0.08 ± 0.01				0.13	
28	1381	α-Ylangene/SH			0.26 ± 0.11					
29	1389	β-Bourbonene/SH			0.15 ± 0.03					
30	1393	Unidentified		0.21 ± 0.03						
31	1402	Unidentified	0.23 ± 0.07							
32	1419	β-Caryophyllene/SH	4.54 ± 0.23	3.28 ± 0.21	5.66 ± 0.22	0.58			3.76	2.89
33	1429	Unidentified	0.39 ± 0.01	0.29 ± 0.3						
34	1432	γ-Cadinen/SH			0.24 ± 0.09					
35	1439	α-Guaiene/SH			0.14 ± 0.01					
36	1456	α-Humulene/SH	4.89 ± 021	4.29 ± 0.21	8.64 ± 0.25	0.94		1.45		1.47
37	1479	γ-Muurolene/SH	0.40 ± 0.08	0.26 ± 0.02	0.63 ± 0.06					
38	1500	α-Muurolene/SH			0.43 ± 0.07					
39	1512	Unidentified			0.06 ± 0.01					
40	1523	δ-Cadinene/SH	0.10 ± 0.01	0.03 ± 0.01	0.17 ± 0.01					
41	1576	Isoledene/SH			0.61 ± 0.05					
42	1583	Caryophyllene oxide/SO	0.19 ± 0.01	0.29 ± 0.03	0.22 ± 0.07					
43	1592	Viridiflorol/SO			3.09 ± 0.19					0.6
44	1593	Unidentified	2.34 ± 0.19	1.88 ± 0.18						
45	1594	Unidentified	0.35 ± 0.03	0.61 ± 0.08	0.6 ± 0.09					
46	1603	Unidentified			0.08 ± 0.01					
47	1607	Unidentified			0.6 ± 0.08					

MH—monoterpene hydrocarbons; MO—oxygenate monoterpene; SH—sesquiterpene hydrocarbons; SO—oxygenated sesquiterpene, KI—Kovats retention index.

**Table 2:** The ATR-FTIR absorption band for SEO samples and vibrational assignments.

Wavenumber (cm^−1^) of ATR-FTIR Recorded Bands			Vibrational Assignment
L-SEO	B-SEO	EG-SEO	
3459	3474	3474	(O-H) stretching vibration [57]
3066	3058	3058	(CH) stretching vibration of CH_3_ and CH_2_ (Csp^3^ and Csp^2^) [57]
2951	2958	2958	
2926	2929	2930	
2875	2875	2875	
1741	1741	1741	(C=O) stretching vibration in carbonyl group; (C=C) stretching vibration in (>C=CH_2_), (-CH=CH-), and (-CH=C<) alkyl groups [20,56,57,58,59]
1642	1637	1638	
1456	1455	1455	(C-H) symmetric and asymmetric bending vibration of (CH_3_) and (CH_2_) groups, (C-H) in-plane bending, (C-O) symmetric and asymmetric stretching vibration, (O-H) in-plane bending, (CH_3_(CO)) symmetric bending, (C-O-C) symmetric and asymmetric stretching [20,57,59,60]
-	1415	1415	
1371	1371	1371	
1303	1303	1303	
1266	1274	1274	
1236	1241	1241	
1214	1215	1215	
1166	1165	1165	
1106	1104	1104	
1080	1077	1078	
1054	-	-	
-	1045	1045	
1022	1022	1022	
982	983	983	(CH_2_) and (CH) out-of-plane wagging, (O-H) out-of-plane banding vibration [57,59,60,61]
877	880	879	
844	847	848	
817	810	810	
759	750	750	
642	642	642	

**Table 3:** Patients’ demographic and clinical characteristics.

Variable	Control Group	Salvia EO Group	Total	*p*-Value
	n (%)			
Sex (Female/Male)	14/36	46/78	174 (100)	
Weight (Kg)	76.80 ± 13.74	74.98 ± 14.74		0.2173 *
**Educational level**				0.5877 **
Middle school	7 (14)	11 (9)	18 (10)	
High school	25 (50)	68 (55)	93 (53)	
University	18 (36)	45 (36)	63 (36)	
**Social status**				0.3032 **
Social aid	3 (6)	7 (6)	10 (17)	
Active	24 (48)	75 (60)	99 (57)	
Retired	23 (46)	42 (34)	65 (37)	
**Health status in the last year**				0.2839 **
Excellent	13 (26)	34 (27)	47 (27)	
Very good	5 (10)	24 (19)	29 (17)	
Good	17 (34)	36 (29)	53 (30)	
Bad	15 (30)	26 (21)	41 (24)	
Very bad	0 (0)	4 (3)	4 (2)	
**Do you use fragrances/aroma in rooms/cars?**				0.1135 **
Daily	22 (44)	63 (51)	85 (49)	
1–3 times/week	5 (10)	23 (19)	28 (16)	
No	23 (46)	38 (31)	61 (35)	
**Do you use perfumes?**				0.4354 **
Daily	18 (36)	57 (46)	75 (43)	
1–3 times/week	22 (44)	49 (40)	71 (41)	
No	10 (20)	18 (15)	28 (16)	
**Do you smoke?**				0.0046 **
Daily	7 (14)	46 (37)	53 (30)	
Occasionally	18 (36)	42 (34)	60 (34)	
No	25 (50)	36 (29)	61 (35)	
**Do you drink alcoholic drinks?**				0.1561 ***
Daily	0 (0)	1 (1)	1 (1)	
Occasionally	10 (20)	51 (41)	61 (35)	
No	40 (80)	72 (58)	112 (64)	
**Do you use sedatives/anxiolytics?**				0.0951 ***
Daily	0 (0)	0 (0)	0 (0)	
Occasionally	15 (30)	31 (25)	46 (26)	
No	35 (70)	93 (75)	128 (74)	
**Do you suffer from a chronic disease?**				0.6036 ****
Yes	19 (38)	42 (34)	61 (35)	
No	31 (62)	82 (66)	113 (65)	
**Do you suffer from any drug/food allergies?**				0.0186 ****
Yes	9 (18)	46 (37)	55 (32)	
No	41 (82)	78 (63)	119 (68)	

n—number of patients; * *t*-test; ** chi-squared test; *** Kruskal-Walis test; **** Fisher’s exact test.

## Data Availability

Data is contained within the article.
